# Crossover frequencies in spermatocytes of Robertsonian homozygotes and heterozygotes of *Mus musculus domesticus*

**DOI:** 10.1590/1678-4685-GMB-2024-0219

**Published:** 2025-08-01

**Authors:** Eliana Ayarza, Marisel González, Jesús Page, Soledad Berríos

**Affiliations:** 1 Universidad de Chile, Instituto de Ciencias Biomédicas, Facultad de Medicina, Programa de Genética Humana, Santiago, Chile.; 2 Universidad de Chile, Facultad de Medicina, Departamento Tecnología Médica, Santiago, Chile.; 3 Universidad Autónoma de Madrid, Departamento de Biología, Edificio de Ciencias Biológicas, Cantoblanco, Madrid, Spain.

**Keywords:** Meiosis, Robertsonian chromosomes, crossovers, Mus musculus domesticus, spermatocytes

## Abstract

Crossovers (COs) generate genetic diversity and proper homologous chromosome segregation during meiosis. *Mus musculus domesticus*, with a diploid number of 2n=40, has 19 autosomal pairs plus one sex chromosome pair all of which are telocentric chromosomes. Frequently exhibits Robertsonian fusions (Rb), which create natural populations with reduced chromosome numbers according to the Rb chromosomes. We examined the number and distribution of COs in spermatocytes from standard homozygous 2n=40 individuals, compared to homozygous Rb 2n=24 and heterozygous Rb 2n=32 individuals carrying 8 trivalents. Spermatocyte nuclear spreads from homozygous and heterozygous were prepared, and immunocytochemistry was used to detect the MLH1 protein for crossover (CO) and the SYCP3 protein for synaptonemal complexes in bivalents or trivalents. We observed an average of 26 ± 2.1 COs in 2n=40, 20.1 ± 1.6 COs in 2n=24, and an intermediate value of 22.4 ± 2.0 COs in 2n=32 spermatocytes. The lower frequency of COs in 2n=24 and 2n=32 spermatocytes compared to 2n=40 may be due to interference from the pericentromeric heterochromatin present in the Rb bivalent or trivalent chromosomes. Additionally, we suggest that the spatial positioning and interactions of these derivative chromosomes in the nucleus could help explain the differences in COs between 2n=24, 2n=32, and 2n=40 spermatocytes.

## Introduction

During meiosis, homologous recombination is a fundamental process that facilitates the exchange of genetic material between parental genomes, ensuring proper chromosome segregation during the first meiotic cell division (Zickler and Kleckner, 2016). This process occurs more frequently in certain regions of the eukaryotic genome known as hotspots (Lichten and Goldman, 1995). Extensive mapping of crossover events in both humans and mice has led to the identification of a family of DNA sequence motifs associated with these hotspots (Myers et al., 2008). These motifs serve as binding sites for the PRDM9 protein, which possesses zinc fingers and a SET histone methyltransferase domain (Baudat et al., 2013). PRDM9 orchestrates meiosis-specific H3K4me3 modifications, rearranging nucleosomes around target motifs and positioning DNA double-strand breaks (DSBs) (Mihola et al., 2009; Youds and Boulton, 2011). The initiation of DSBs in eukaryotes depends on the SPO11 protein (Keeney et al., 1997; Keeney, 2001). DSBs catalyzed by the SPO11 protein trigger a response similar to that initiated by DNA damage, leading to the recruitment of the metabolic machinery and the processing of recombination intermediates. DSB repair can result in either non-crossover (NCO) events, potentially leading to gene conversion, or crossover (CO) events. These recombination events follow distinct repair pathways (Guillon et al., 2005).

Aside from hotspots, various factors influence the distribution of recombination events: (1) each bivalent undergoes at least one crossover event, regardless of its length (Mather, 1936); (2) in addition to this obligatory crossover, the number of additional recombination events is proportional to chromosome length, with longer chromosomes having a higher likelihood of multiple crossovers (Broman et al., 2002); (3) a crossover event in one region of a chromosome reduces the likelihood of another nearby crossover (known as positive interference) (Wu and Lichten, 1994); (4) crossovers usually occur in euchromatin and rarely in heterochromatin, with recombination events near centromeres (pericentromeric heterochromatin) being extremely rare due to centromeric interference (Sherman and Stack, 1995); and (5) the frequency and distribution of recombination events often differ between sexes (Wang et al., 2017).

In the western European subspecies *Mus musculus domesticus*, Robertsonian (Rb) fusions are common chromosomal rearrangements that produce natural populations with karyotypes that vary in diploid number, ranging from the standard 2n = 40 (all telocentric) to 2n = 22 (with nine pairs of metacentrics) (Piálek et al., 2005; Garagna et al., 2014). Rb translocations involve DNA double-strand breaks at the centromeres of two chromosomes, followed by fusion of the long arms of acrocentric and/or telocentric chromosomes, resulting in a metacentric Rb chromosome (Burgoyne et al., 2009). When populations with specific sets of homozygous metacentric chromosomes encounter other populations with either the standard or metacentric karyotypes, a ‘hybrid zone’ is formed (Hauffe et al., 2012). In Rb chromosomes, a lower frequency of recombination events has been inferred from chiasmata observed at metaphase, likely due to enhanced centromeric interference (Dumas et al., 2014). In heterozygous mice, meiosis has shown an intermediate number of recombination events compared to both the standard 2n=40 variant and homozygous Robertsonian variants 2n=22 (Dumas *et al.*, 2014).

Historically, meiotic recombination studies relied on the physical localization of chiasmata (Hulten, 1974) or linkage analysis (Mikawa et al., 1999). The discovery of proteins involved in crossover formation, especially MLH1 and MLH3 in late recombination nodules, enabled direct recombination studies using immunocytological methods (Anderson *et al.*, 1999). MLH1, a mismatch repair protein, is necessary for meiotic recombination in mammals, and MLH1 foci have been shown to mark crossover sites (Baker et al., 1996). Evidence suggests that the number and distribution of MLH1 foci on SCs closely match those of chiasmata on diplotene-metaphase I chromosomes (Anderson *et al.*, 1999).

Crossover distribution along chromosomes is non-random, due to two underlying factors: first, DSBs are not formed randomly, and second, crossover choice is regulated by crossover interference, which tends to ensure that adjacent crossovers on the same chromosome occur at sites further apart than would be expected by chance. Crossover assurance ensures that all chromosomes acquire at least one crossover, known as the obligate crossover (Youds and Boulton, 2011).

Chromosomal rearrangements, such as the Robertsonian fusions in *Mus musculus domesticus* that produce metacentric chromosomes, provide an opportunity to explore crossover location. It is relevant to examine whether the number and location of crossovers are modified based on the chromosomal constitution of spermatocytes, considering that the arms of the metacentric Rb chromosome are homologous to the telocentric chromosomes of the original 2n=40 *Mus* karyotype. Given this chromosomal homology, similar hotspot locations would be expected among the chromosomes. Thus, any observed variations in distribution or quantity may be attributed to other factors involved in spermatocyte organization.

In this study, we examine the number and distribution of COs through MLH1 foci during meiosis in *Mus musculus domesticus* with the standard 2n=40 karyotype (all telocentric chromosomes and 20 bivalents) compared to spermatocytes with derived karyotypes: homozygotes 2n=24 with 8 Rb metacentric bivalents, and heterozygotes 2n=32 with 8 trivalents.

## Material and Methods

The spermatocytes of six three-month-old *Mus musculus domesticus* males were analyzed. Two were homozygotes with a 2n=40 CD1 karyotype with all telocentric chromosomes; two were Milano II 2n=24 with 8 pairs of metacentric Rb chromosomes, 3 pairs of telocentric chromosomes, and the X and Y sex chromosomes; and two were heterozygotes with a 2n=32 karyotype, presenting 8 Rb chromosomes, 22 telocentric chromosomes (16 of which are homologous to the arms of the metacentric Rb chromosomes), and the X and Y sex chromosomes. The chromosome constitution follows the standard 2n=40 karyotype (Capanna & Castiglia, 2004; Piálek et al., 2005). The heterozygote mice were produced by crossing CD1 2n=40 females with Milano II 2n=24 males, or the reciprocal crosses. During meiosis, 2n=40 individuals present 19 telocentric bivalents plus the XY bivalent; the 2n=24 individuals present 8 metacentric Rb bivalents, three telocentric bivalents plus the XY bivalent; and the 2n=32 individuals present 8 trivalents, three telocentric bivalents, and one XY bivalent.

The mice were maintained at 22 ºC with a 12/12-hour light/dark cycle and were fed *ad libitum.* They were sacrificed by cervical dislocation prior to obtaining the testicles.

Procedures involving the use of mice were reviewed and approved by the Ethics Committee of the Faculty of Medicine, Universidad de Chile (Nº CBA #0441), and by the Ethics Committee of the Chilean National Science Foundation (FONDECYT-CONICYT).

### Spreading and immunocytochemistry

Spermatocyte spreads were obtained following the procedure described by Peters et al. (1997). Briefly, a testicular cell suspension in 100 mM sucrose was spread onto a slide dipped in 1% paraformaldehyde in distilled water containing 0.15% Triton X-100, then left to dry for two hours in a humid chamber. The slides were subsequently washed with 0.08% Photoflo (Kodak), air-dried, and rehydrated in PBS. The slides were incubated for 45 minutes at 37 ºC in a humid chamber with the primary antibodies: rabbit anti-SYCP3 1:100 (ab235254) and mouse monoclonal anti-MLH1 1:100 (Abcam, ab14206) (Anderson *et al*., 1999). Next, the slides were incubated for 30 minutes at room temperature with the secondary antibodies: FITC-conjugated goat anti-mouse IgG (1:50) (Sigma) or Texas red-conjugated goat anti-rabbit IgG (1:200) (Jackson ImmunoResearch). To differentially stain the pericentromeric heterochromatin, the spreads were stained in an aqueous solution of 10 ng/ml DAPI (4‘, 6-diamidino-2-phenylindole) for 5 minutes at room temperature and extensively washed in PBS. Slides were mounted in Vectashield (Vector Laboratories).

### Crossover count (CO) and statistical analysis

The number of MLH1 foci present on the SC of the bivalents and trivalents in each of 200 nuclei of mid-to-late pachytene spermatocytes (2n=40, 2n=24, and 2n=32) was counted-one hundred of each of the two animals per karyotype. For each chromosomal constitution, two averages were calculated: one including the XY bivalent, as the synapsis of the XY sex pair during pachytene differs temporally from that of autosomal bivalents.

The frequency of spermatocytes according to the number of COs in *Mus musculus domesticus* (2n=40, 2n=24, and 2n=32) was also estimated.

The normality of the distribution of sample variables was determined using the Kolmogorov-Smirnov test. For variables that showed a non-normal distribution, mean comparisons were made using the non-parametric Wilcoxon signed-rank test for paired samples. A 95% confidence interval was used for both tests, with a significance level of 5% (α=0.05).

## Results

### Crossovers in pachytene spermatocyte spreadings of *Mus musculus domesticus* 2n=40, 2n=24, and 2n=32

**Figure 1 -:**
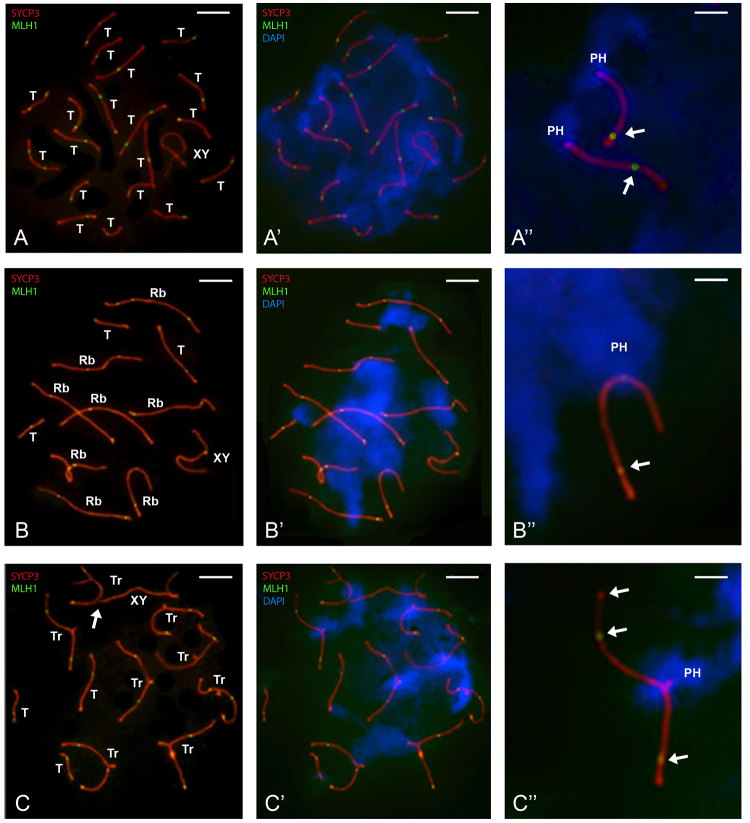
Crossovers (COs) in spreads of spermatocytes with A) 2n=40, B) 2n=24, and C) 2n=32 chromosomes. Synaptonemal complexes (SCs) were labeled with antibodies against SYCP3 (red), COs with antibodies against MLH1 (green), and pericentromeric heterochromatin (PH) was stained with DAPI (blue). Scale bar = 10 μm. **A)** SCs of the 19 telocentric autosomal bivalents (T) and partially synapsed sex chromosome axes forming the XY bivalent (XY) are visible. **A’)** Same nuclear spread showing PH around proximal telomeres in all bivalents (blue). **A’’)** Detail showing SCs of two telocentric bivalents with PH around the proximal telomere and a CO near the distal telomere (arrow). Scale bar = 3 μm. **B)** Eight metacentric bivalents (Rb), three telocentric bivalents (T), and partially synapsed sex chromosome axes forming the XY bivalent (XY) are shown. **B’)** Same nuclear spread, with PH located medially in Rb bivalents and at the proximal telomere in the three telocentric bivalents (blue). **B’’)** Detail of one Rb bivalent with three COs-two within one arm and the third on the opposite arm, closer to the distal telomere. Each telocentric bivalent contains one CO near the distal telomere (arrows). Scale bar = 3 μm. **C)** Eight trivalents, three telocentric bivalents (T), and partially synapsed sex chromosome axes forming the XY bivalent (XY) are shown. The X chromosome’s single axis is bound to a telocentric chromosome axis in an unsynapsed trivalent (arrow). Most trivalents show two COs, while each telocentric and XY bivalent has just one. **C’)** Same nuclear spread showing PH located at the confluence of three centromeric regions within each trivalent and associated with each other. In the three telocentric bivalents, one CO is observed at the proximal telomere. **C’’)** Detail showing the SC of one trivalent with three COs-two within one arm and the third near the distal telomere in the other arm (arrows). Scale bar = 3 μm.

Through immunofluorescent labeling of the MLH1 protein, crossovers (COs) were observed as foci distributed along the synaptonemal complexes (SC) and spanning the width of this structure. COs appeared as yellow fluorescent foci due to the overlap between green (FITC) staining for MLH1 and red (Texas-red) staining for SC. Figure 1 shows representative nuclear spreads and MLH1 foci in the spermatocytes analyzed here. In the 2n=40 spermatocytes, all 19 telocentric autosomal bivalents displayed complete synapsis, as evidenced by the SCs, while the X and Y chromosomes exhibited partial synapsis (Figure 1 A ). In 2n=24 spermatocytes, complete synapsis was observed in the 8 Rb metacentric bivalents and the 3 telocentric bivalents. The X and Y chromosomes displayed partial synapsis (Figure 1 B ). In 2n=32 spermatocytes, eight trivalents were formed, each consisting of an Rb metacentric chromosome synapsed with two telocentric chromosomes homologous to its arms. Complete synapsis was observed in the three telocentric bivalents, with partial synapsis between the X and Y chromosomes (Figure 1 C ). In all the studied nuclear microspreads, bivalents and trivalents showed several COs, most of which were localized near the telomeric regions of the SCs (Figure 1 C”). In some cases, the synaptic region between the X and Y chromosomes did not exhibit a CO (Figure 1 A ).

When the nucleus is disrupted by spreading procedures, its three-dimensional architecture is lost; however, remnants of its previous organization can still be observed. Among these, the most prominent is the association between different types of bivalents through pericentromeric heterochromatin, which is abundant in all bivalents of *Mus musculus domesticus.* In 2n = 40 spermatocytes, various groups of telocentric bivalents were associated through pericentromeric heterochromatin (Figure 1 A’ ). In contrast, in 2n = 24 spermatocytes, depending on the specific bivalents involved, we observed associations between metacentric Rb bivalents, as well as associations among the three telocentric bivalents, all mediated by heterochromatin (Figure 1 B’ ). In 2n = 32 spermatocytes, trivalents were grouped in associations of two or three, connected via pericentromeric heterochromatin. Additionally, in cases where the short arms of the telocentric chromosomes did not synapse, these regions often associated with the asynaptic axes of other trivalents or with the asynaptic axis of the XY bivalent (Figure 1 C, arrow).

Across all karyotypes, most telocentric bivalents exhibited a crossover (CO) near the distal telomere (Figures 1 A” , 1B”, 1C”). Rb bivalents displayed one or two COs, occasionally three, typically located on both chromosome arms near the distal telomeres (Figures 1 B’  and 1B”). Trivalents exhibited two or three COs on different arms, mainly near the distal telomeres, with some located toward the middle of a chromosome arm (Figure 1 C” ).

### Number of crossovers (CO) per spermatocyte in pachytene of *Mus musculus domesticus* 2n=40, 2n=24, and 2n=32

**Figure 2 -:**
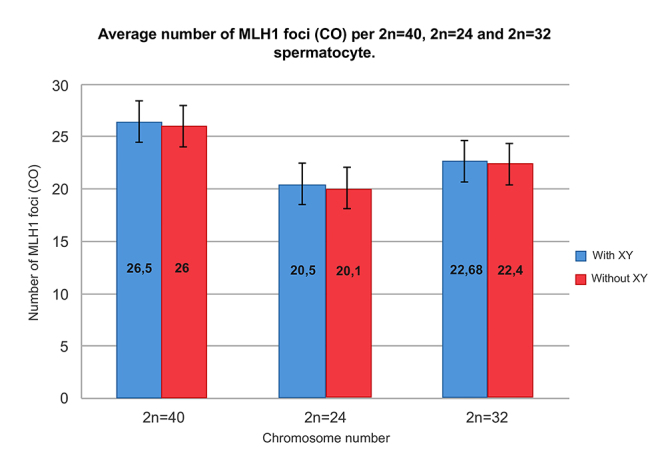
Average number of MLH1 foci (CO) per spermatocyte for 2n=40, 2n=24, and 2n=32 chromosome complements. Blue bars represent the average number of COs including the XY bivalent, while red bars represent the average excluding it. A significant difference exists in the average CO number between 2n=40 and both 2n=24 and 2n=32 spermatocytes. There is also a significant difference between 2n=24 and 2n=32 spermatocytes (Wilcoxon signed-rank test).

The number of MLH1 foci present on the SC of the bivalents and trivalents in one hundred of each of the two animals nuclei of mid-to-late pachytene spermatocytes with karyotypes 2n=40, 2n=24, and 2n=32 was scored. This analysis revealed distinct crossover (CO) profiles associated with each karyotype. Overall, spermatocytes from individuals with 2n=40 chromosomes exhibited the highest average number of COs, followed by 2n=32 and then 2n=24. This trend was consistent whether the foci on the XY bivalent were included or excluded. No significant differences were detected between individuals sharing same karyotype (Figure 2; Table 1).

**Table 1 -:** Average number of MLH1 foci (CO) per 2n=40, 2n=24 and 2n=32 spermatocytes.

Chromosome number	Number of spermatocytes	Average CO per spermatocyte with XY	Average CO per spermatocyte with XY	Average CO per spermatocyte without XY	Average CO per spermatocyte without XY
2n=40 A	100	27.09 ± 2.10	26.5 ± 2.2	26.56 ± 2.10	26.0 ± 2.1
2n=40 B	100	25.91 ± 2.05		25.50 ± 1.95	
2n=24 A	100	20.36 ± 2.13	20.5 ± 1.9	20.04 ± 1.90	20.1 ± 1.6
2n=24 B	100	20.63 ± 1.56		20.22 ± 1.35	
2n=32 A	100	22.97 ± 2.15	22.68 ± 2.2	22.58 ± 1.97	22.4 ± 2.0
2n=32 B	100	22.39 ± 2.23		22.15 ± 2.10	

The presence of at least one MLH1 focus in the short homologous region between the X and Y chromosomes in most spermatocytes was consistent with the required chiasmatic connection necessary for their proper segregation. However, since MLH1 was not always detected in the synaptic region between the X and Y chromosomes (Figure 1 A ), crossover (CO) counts per spermatocyte were analyzed both with and without including the XY bivalent (Table 1).

A non-parametric Wilcoxon test confirmed that the differences in CO numbers among all three karyotypic groups were statistically significant (p < 0.0001 for all pairwise comparisons), regardless of whether the XY bivalent was included. These findings suggest a potential relationship between chromosome number and recombination frequency in *M. m. domesticus* (Figure 2; Table 1).

**Figure 3 -:**
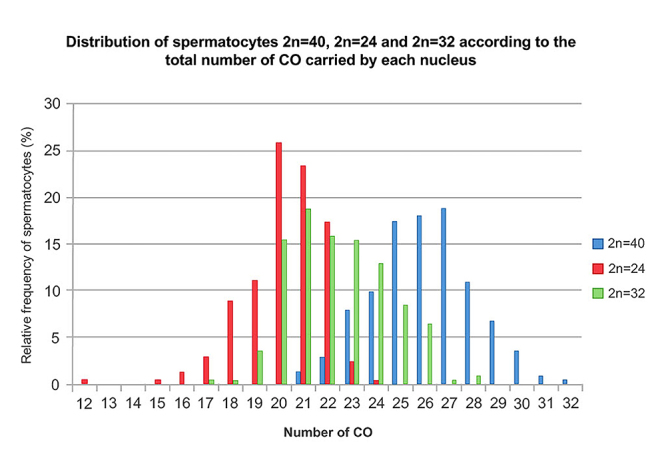
Distribution of spermatocytes with 2n=40, 2n=24, and 2n=32 chromosomes based on the total number of COs per nucleus. The 2n=40 spermatocytes (blue bars) have COs distributed between 20 and 30, while the majority of 2n=24 spermatocytes (red bars) range between 15 and 25 COs. The 2n=32 spermatocytes (green bars) have COs distributed between 20 and 27, positioning them between the other two groups.

To assess statistical significance between the average COs per spermatocyte in the different karyotypes, the non-parametric Wilcoxon test was used due to the non-normal distribution. Significant differences were found in the average number of COs per spermatocyte across all comparisons: 2n=40/2n=24, 2n=40/2n=32, and 2n=24/2n=32 (p < 0.0001 at the 5% significance level).

### Frequency of pachytene spermatocytes relative to crossovers (CO) in *Mus musculus domesticus* 2n=40, 2n=24, and 2n=32

Spermatocytes of each karyotype were grouped according to their total number of crossovers (COs) per nucleus. The Kolmogorov-Smirnov test, applied to each dataset, indicated that the distribution of each population was non-normal (p < 0.0001 at the 5% significance level) revealing three partially overlapping curves. Spermatocytes from 2n=40 karyotype had the highest CO values, and those from 2n=24 the lowest, and those from 2n=32 showed intermediate values (Figure 3; Table 2).

**Table 2 -:** Distribution of spermatocytes 2n=40, 2n=24 and 2n=32 according to the total number of CO carried by each nucleus.

***Mus domesticus* 2n=40**	Number of COs	21	22	23	24	25	26	27	28	29	30	31	32	
	Number of spermatocytes	3	6	16	20	35	36	38	22	14	7	2	1	
	Relative frequency (%)	1.5	3	8	10	17.5	18	19	11	7	3.5	1	0.5	
***Mus domesticus* 2n=24**	Number of COs	12	13	14	15	16	17	18	19	20	21	22	23	24
	Number of spermatocytes	1	0	0	1	3	6	18	31	52	47	35	5	1
	Relative frequency (%)	0.5	0	0	0.5	1.5	3	9	11	26	23.5	17.5	2.5	0.5
***Mus domesticus* 2n=32**	Number of COs	17	18	19	20	21	22	23	24	25	26	27	28	
	Number of spermatocytes	1	1	7	31	38	32	31	26	17	13	1	2	
	Relative frequency (%)	0.5	0.5	3.5	15.5	19	16	15.5	13	8.5	6.5	0.5	1	

In 2n=24 spermatocytes, the total number of CO ranged from 12 to 24. Within this group, 26% of cells displayed 20 COs, and 67% exhibited between 20 and 22 COs (Figure 3; Red Bars).

In 2n=32 spermatocytes, the CO count ranged from 17 to 28, with 19% of spermatocytes showing 21 COs, and 63.5% sfalling within the range of 21 to 24 COs (Figure 3; Green Bars).

In 2n=40 spermatocytes, COs value ranged from 21 to 32. A subset of 19% of spermatocytes had 27 COs, while 65.5% exhibited between 25 and 28 COs (Figure 3; Blue Bars).

## Discussion

The molecular understanding of crossover (CO) formation has advanced significantly; however, the mechanisms that govern CO distribution along meiotic chromosomes remain unclear (Youds and Boulton, 2011; Marín-García et al., 2024). Chromosomal rearrangements can contribute to reproductive isolation by affecting large genomic regions, majorly due to the absence of homologous recombination (Rieseberg, 2001; Butlin, 2005). Although empirical and theoretical studies on recombination suppression have often focused on inversions and reciprocal translocations (Faria and Navarro, 2010; Fishman et al., 2013), the Robertsonian (Rb) fusion/fission, a more common rearrangement, has received relatively little attention (King, 1993). Prior studies have shown reduced CO rates in homozygous Rb mice compared to standard mice in the centromeric regions (Castiglia and Capanna, 2002; Dumas and Britton-Davidian, 2002; Bidau et al., 2011; Merico et al., 2013; Dumas *et al.*, 2014). Recombination rates in wild Rb heterozygotes have also been examined but often involve polymorphic individuals with varying numbers of Rb bivalents and trivalents, complicating the assessment of each meiotic configuration’s effect (Wallace et al., 1992; Castiglia and Capanna, 2002; Bidau *et al.*, 2011; Capilla et al., 2014). In contrast, detailed studies in single-Rb heterozygotes have shown varying degrees of recombination suppression, suggesting an influence of genetic background (Dumas *et al.*, 2014).

In this study, the total CO count in 2n=24 individuals homozygous for 8 metacentric Rb chromosomes was lower than in 2n=40 individuals, while the average CO count in 2n=32 hybrid spermatocytes was intermediate, similar to previous findings in homozygous Rb individuals with 2n=24 and 2n=22 (Dumas & Britton-Davidian, 2002; Bidau et al., 2011; Dumas *et al.*, 2014). When spermatocytes were grouped by CO count, the distribution curve for 2n=32 was intermediate, with 2n=24 spermatocytes exhibiting fewer COs compared to 2n=32 or 2n=40. In all cases, Rb homozygotes showed fewer COs in proximal regions than distal regions, consistent with prior reports (Dumas and Britton-Davidian, 2002). This reduction in proximal recombination may be associated with centromeric interference, potentially accentuated in Rb metacentric chromosomes (Anderson *et al.*, 1999; Merico et al., 2013; Dumas and Britton-Davidian, 2002; Dumas *et al.*, 2014). However, this does not fully explain the higher CO counts in trivalents, whose pericentromeric heterochromatin quantity does not differ markedly from that of Rb bivalents. On the contrary, it could be thought that in trivalents it is relatively more abundant considering the structure of the participating chromosomes. In metacentric Rb bivalents, two duplicated Rb chromosomes are found in synapse, each of them the product of chromosomal fusion of two original telocentric chromosomes with the loss of their short arms and part of their heterochromatin. In each trivalent, however, a duplicated metacentric Rb chromosome synapses with the two duplicated telocentric chromosomes that are homologous to its arms and have completely retained their pericentromeric heterochromatin (Garagna et al., 2014).

Synapsis around the centromere and pericentromeric heterochromatin is crucial in heterozygous trivalent configurations in the house mouse. Most trivalents achieve complete synapsis between the heterologous short arms of telocentric chromosomes and between centromeres and arms of telocentrics with those of metacentrics (Figure 1 C ). However, open configurations with unsynapsed telomeric ends od telocentric chromosomes are also common, allowing heterologous associations with other chromosomes. These associations can form between single chromosomal axis of trivalents in open configurations or with sex chromosomes single axis, potentially interfering with CO formation and normal meiotic progression (Manterola et al., 2009; Vasco et al., 2012). Other studies on the germinal epithelium of Rb heterozygotes with 2n = 31 or 2n=32 have revealed a distortion in the typical 1:4 ratio between spermatocytes and spermatids, due to a marked reduction (~66%) in the number of spermatids compared to *Mus m.* domesticus 2n = 40 (Garagna et al., 2001; González et al., 2015). Additionally, caspase-3-positive apoptotic cells were significantly more abundant in heterozygotes than in parental homozygotes, particularly at stage XII of the seminiferous epithelium, which corresponds to meiotic metaphase spermatocytes. The increased apoptosis among dividing spermatocytes in Rb heterozygotes likely reflects the selective elimination of cells with chromosomal misalignment or segregation defects. This is consistent with the lower spermatid count and reduced fertility observed in multiple Rb hybrids (González *et al.*, 2015; Manieu et al., 2014).

Studies have shown that the number and distribution of early recombination nodules (RPA foci) are similar in Rb and 2n=40 spermatocytes, suggesting that COs are likely redistributed during nodule resolution rather than double-strand break (DSB) formation (Capilla et al., 2014). These observations suggest that telocentric and metacentric Rb chromosomes may contain similar sequences involved in DSB formation, but only some are later transformed into COs. CO positions may vary due to genetic interference, largely determined by abundant pericentromeric heterochromatin in *M. m. domesticus* spermatocytes, leading to distal telomeric localization in both telocentric and Rb bivalents (Merico et al., 2003; Berríos, 2017). On the other hand, considering the genetic background of the individuals studied, one might have expected the hybrids to be the most genetically divergent compared to homozygotes, particularly considering the possible diversity of Prdm9 alleles. This could have eventually led to the hotspots between the participating chromosomes in trivalent not matching or not leading to effective crossovers (Smagulova et al., 2016). However, subsequent works have shown that the advantage of new PRDM9 alleles is in limiting the number of binding sites used effectively, rather than in increasing net PRDM9 binding, suggesting that the evolutionary advantage of hotspots may have been to increase the efficiency of DSB repair and/or homolog pairing (Baker *et al.,* 2023; Genestier et al., 2024). In this analysis, another perspective that we believe is important to consider is that the chromosome reorders impact the global architecture of the nucleus in prophase I of meiosis, influencing the possible relationships among chromosomal and nuclear domains (Berríos *et al.*, 2010; Berríos, 2017; Marín-García et al., 2024). It is so that the presence of Rb metacentric bivalents determines the nuclear architecture of 2n = 24 spermatocytes, generating two main nuclear domains. One is located toward the center of the nucleus, containing fewer chromocenters, each formed by the association of pericentromeric heterochromatin from two or more Rb bivalents. The second is positioned at the nuclear periphery, where the three telocentric bivalents associate via their pericentromeric heterochromatin, which is also anchored to the nuclear envelope (Berríos *et al.*, 2014).

In 2n = 32 heterozygotes, these two domains are not established. Instead, trivalents and telocentrics are dispersed throughout the nuclear periphery. In trivalents, the pericentromeric heterochromatin of the metacentric Rb bivalent and that of the corresponding telocentric bivalents are spatially associated at the nuclear periphery and are tightly bound to the nuclear envelope. Although the spreading procedure disrupts the nucleus and results in the loss of its three-dimensional structure, traces of its original organization remain detectable. One of the most notable features is that it is possible to appreciate the various types of associations according to the different bivalents or trivalents through the abundant pericentromeric heterochromatin of all the chromosomes of *Mus m domesticus.*

The abundant accumulation of heterochromatin could potentially interfere with crossover (CO) formation. However, we observed that the number of COs in trivalents was higher than in the metacentric Rb bivalents of 2n = 24 homozygous spermatocytes. While we do not rule out a possible interference effect of heterochromatin on CO formation, we propose that the pericentromeric heterochromatin accumulations at the nuclear periphery may exert a milder interference effect. Telocentric bivalents and trivalents positioned at the nuclear periphery may gain stability, favoring telomeric CO localization near the double-strand break (DSB) repair machinery. This spatial arrangement may facilitate the proper assembly of the recombination machinery on homologous chromosomes (Merico et al., 2003). Consequently, the DNA sequences prone to DSBs on each chromosome may influence CO formation.

Taken together, the interactions among chromosomal domains-such as pericentromeric heterochromatin associations-may significantly affect nuclear bivalents or trivalents distribution, associations between chromosomal domains, and CO establishment.

## Data Availability

The entire dataset supporting the results of this study was published in the article itself.
